# Analysis of the Mechanical Properties of a Stabilized Subgrade Type Soil under a Sustainable Approach for Construction

**DOI:** 10.3390/ma16196395

**Published:** 2023-09-25

**Authors:** Pedro Gallego-Quintana, Omar Farid Ojeda-Farias, Alexander Alvarez-Rosario, Ervin Jesús Alvarez-Sánchez, Laura Landa-Ruiz, Bernardo T. Terán-Torres, José Manuel Mendoza-Rangel, Miguel Ángel Baltazar-Zamora

**Affiliations:** 1 Facultad de Ingeniería Mecánica Eléctrica Xalapa—FIME, Universidad Veracruzana, Circuito G. Aguirre Beltrán S/N, Lomas del Estadio, Xalapa 91000, Veracruz, Mexico; pedro.gallegoq@ucc.edu.co (P.G.-Q.); eralvarez@uv.mx (E.J.A.-S.); 2 Programa de Ingeniería Civil, Facultad de Ingeniería, Universidad Cooperativa de Colombia, Sede Ibagué-Espinal, Cll. 10 1-64, Ibagué 0138, Tolima, Colombia; alexander.alvarezr@campusucc.edu.co; 3 Facultad de Ingeniería Civil, Universidad Autónoma de Nuevo León, Av. Universidad S/N, Ciudad Universitaria, San Nicolás de los Garza 66455, Nuevo León, Mexico; omar.ojedafrs@uanl.edu.mx (O.F.O.-F.); bernardo.terantrr@uanl.edu.mx (B.T.T.-T.); 4 Facultad de Ingeniería Civil-Xalapa, Universidad Veracruzana, Lomas del Estadio S/N, Zona Universitaria, Xalapa 91000, Veracruz, Mexico; lalanda@uv.mx

**Keywords:** unpaved roads, sugarcane bagasse ash, agro-industrial waste management, roads

## Abstract

This article presents an experimental study to analyze the mechanical properties of a soil stabilized with ordinary Portland cement (OPC) under a sustainable approach consisting of a significant substitution of OPC for sugarcane bagasse ash (SCBA) to reduce the quantity of cement used in the stabilization, reaching the necessary mechanical requirements for its use as a subgrade layer. Soil specimens were elaborated with 3%, 5%, and 7% OPC as a stabilizing agent by weight of the soil. These mixtures were then partially substituted with 25%, 50%, and 75% SCBA, with these percentages being by weight of the stabilizer (OPC). Compaction, compressive strength, and California bearing ratio (CBR) tests were performed to evaluate the mechanical properties of the specimens. The results indicate that a 25% substitution of OPC by SCBA shows a similar performance to the mixture with only Portland cement, so a reduction in OPC use can be made. Further, with a substitution of 100% OPC by SCBA, the CBR of natural soil without stabilizers is improved.

## 1. Introduction

Developing stabilized soils under a sustainable approach requires reducing the use of ordinary Portland cement (OPC) and the overexploitation of material banks [1]. Stabilizing floors with cement involves mixing both materials in specific proportions to create a homogeneous and resistant mixture [2,3]. However, the use of cement in this technique generates a considerable environmental impact since its production is one of the sources of carbon dioxide emissions (CO_2_) [4,5]. Additionally, although cement provides resistance to the stabilized soil, it can generate thermal expansion and contraction during its hydration. In this sense, the environmental and economic crisis that our surroundings are experiencing have forced a change in approaches such as soil stabilization, using materials that seek to improve their quality and resistance for use in construction, with a low impact on their carbon footprint and the optimization of natural resources [6,7,8,9]. Among the materials that meet these characteristics are the waste and by-products of the industry, such as ashes, slag, sludge, coal gangue, glass powder, and residues from chemical processes, among others [10,11,12,13].

In the context of soil stabilization using plant-based ashes, sugarcane bagasse ash is not the only alternative that has been studied. Contemporary research underscores the potential of these ashes, demonstrating significant efficacy in augmenting the geotechnical attributes of soils, particularly when incorporated into road subgrades. Cherian and Siddiqua (2021) explored the potential of recycled pulp mill fly ash (PFA) as a hydraulic binder for stabilizing weak silty sand subgrades [7]. Their findings revealed that PFA treatment considerably improved the soil’s physicochemical, mechanical, and ecotoxicological attributes. The enhanced strength and stiffness of the soil, even after short-term curing, were attributed to the hydration reactions of calcium-rich PFA, leading to the formation of stable cementitious compounds. Silva et al. (2023) investigated rice husk ash (RHA) as a stabilizing additive for lateritic soil [14]. Their research emphasized the combined effect of RHA and phosphoric acid on the soil’s resilient behavior and compressive strength. The optimal results were achieved with a 10% RHA and 5% phosphoric acid composition. In a distinct study, Fadmoro et al. (2021) employed mixed cow dung and husk ashes for soil stabilization [15]. Their results indicated significant improvements in soil parameters, especially in the California bearing rratio (CBR) value, with the inclusion of 20% ash. Adeyanju et al. (2020) integrated a rice husk ash-based geopolymer (GRHA) with cement kiln dust (CKD) for soil stabilization [16]. Their findings underscored the superior efficacy of an RHA-based geopolymer over CKD in enhancing the soil’s mechanical properties. Furthermore, recent studies have also spotlighted coffee husk ash as a promising material for soil stabilization [17,18,19]. These investigations collectively suggest that the addition of coffee husk ash to soils, especially expansive ones, can significantly improve their geotechnical properties. In conclusion, plant-based ashes, derived from various sources, present a sustainable and eco-friendly alternative for soil stabilization in road construction. Their incorporation not only enhances the geotechnical properties of soils but also offers a viable solution for waste management.

Sugarcane bagasse ash (SCBA) is a by-product of the sugar industry that is produced during the burning of bagasse [20]. Its availability or production in only three Latin American countries is 0.031 Gt annually, representing 80.51% of the worldwide production [21]. This by-product has been used as a supplementary cementing material in concrete and mortar mixes due to its pozzolanic reactivity between its aluminosilicates and the calcium hydroxide or portlandite Ca(OH)_2_ produced during the hydration phase [22,23,24,25,26]. However, its characteristics allow its use as a stabilizing agent that can partially replace cement. Different studies have analyzed the feasibility of its use by combining SCBA and OPC for soil stabilization [27]. This combination leads to an improvement in soil properties compared to the use of either material separately. In reference [28], the use of rice husk ash and SCBA to improve the properties of low-plasticity clayey soils was investigated, with potential applications as a subgrade. The main result was that a mixture of 5% of each ash type increased the California bearing ratio (CBR) value of the soil from 12% to 33.75%, significantly improving its load capacity and reducing its optimum moisture content. Thus, the potential of these agricultural by-products in soil improvement was established.

On the other hand, reference [29] sought to develop alternative and ecological cementing agents using SCBA to stabilize highly fertile organic soils. The authors found that the stabilized soil mixture where the OPC was partially replaced with percentages of 20% and 5% SCBA reached an unconfined compressive strength (UCS) that exceeded the reference samples. In the same way, in reference [30], a soil was stabilized for subgrade using rice husk ash (RHA), SCBA, and cow dung ash (CDA). The ashes were mixed with the soil by partial substitution by weight in proportions of 0, 2.5, 5, 7.5, 10, and 12.5%, and it was found that the optimal proportion of ash was 7.5%, achieving significant improvements in the CBR and UCS.

Despite the existence of a large amount of research that proves the effectiveness of SCBA in soil stabilization, studies that delve into the behavior of soils stabilized with SCBA using test methods that are employed in the design and construction of roads with a sustainability approach seeking to reduce the amount of OPC are still required. Under this approach, the construction of communication routes will have a low carbon footprint, becoming economically viable and socially responsible.

Accordingly, the aim of this study was to analyze the mechanical properties of a soil stabilized with OPC under a sustainable approach consisting of a significant substitution of OPC by SCBA to reduce the amount of cement used in stabilization, reaching the mechanical requirements necessary for its use as a subgrade layer.

For this purpose, soil specimens were elaborated with 3%, 5%, and 7% OPC as a stabilizing agent by weight of the soil. These mixtures were then partially substituted with 25%, 50%, and 75% SCBA, with these percentages being by weight of the stabilizer (OPC). The microstructural characteristics of the soil, the OPC, and the SCBA were obtained. Subsequently, the mechanical properties of the specimens, including compaction, compressive strength, and the California bearing ratio, were analyzed during the experimentation phase.

## 2. Materials and Methods

### 2.1. Materials

The soil used in this study was extracted by manual exploration in the field within the rural private farm Monte Madero (N2098210,689683 E4756222,043045) located at 3200 m a.s.l., 4 km from the urban area of the Municipality of Murillo, Tolima, Colombia, and beside the Cambao-Manizales secondary road. The geotechnical properties of this soil were obtained through laboratory tests carried out according to the procedures established in the American Society for Testing and Materials (ASTM) standards [31,32,33,34,35].

The materials used as soil stabilizing agents were OPC type I [36] and SCBA, the latter being obtained from the sugarcane mill Trapiche Industrializado El Escobal in the Municipality of Ibagué, Tolima, Colombia. The SCBA was obtained from one of the two boilers, which reach a combustion temperature of up to 700 °C. Following combustion, the SCBA was carefully extracted, packaged, and stored in sealed containers to prevent contamination during transportation and handling. The ash underwent an ambient air-drying process, followed by sieving to remove larger particles and any residual debris. It is important to note that the ash was not subjected to any additional chemical or physical treatments, ensuring its representation in real-world scenarios as a raw material or input. The SCBA was stored in airtight containers to avoid contamination.

**Figure 1:**
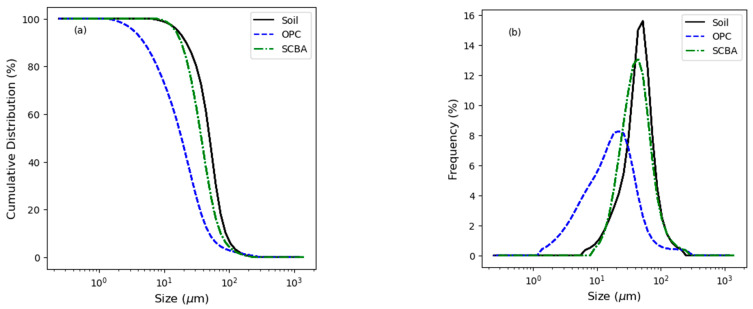
Particle size distribution of the soil (D_50_ = 50.20 µm), the ordinary Portland cement (OPC) (D_50_ = 18.56 µm), and the sugarcane bagasse ash (SCBA) (D_50_ = 37.43 50.20 µm) used in this study. (**a**) Cumulative distribution (%) and (**b**) frequency distribution (%).

Table 1 shows the geotechnical properties of the soil used in this study, while Table 2 presents the chemical composition of the soil, the OPC, and the SCBA obtained by X-ray fluorescence (XRF) with an Epsilon 3 PANalytical (Malvern, UK) analyzer. SCBA has a content higher than 70% in the sum of the main oxides of a pozzolanic material (SiO_2_, Al_2_O_3_, and Fe_2_O_3_) [37]. Figure 1 shows the particle size distribution of the soil, the OPC, and the SCBA obtained by laser ray diffraction in a MICROTRAC S3500 analyzer (Microtrac MRB, Montgomeryville and York, PA, USA). In all cases, the materials used passed through a 0.075 mm mesh. In the accumulated and frequency distributions, the OPC presented a higher fineness with an average diameter of 26.51 µm, followed by the SCBA with 43.96 µm and, finally, the soil, with the largest average diameter of 54.76 µm. The frequency distribution shows that both OPC and SCBA have a variety of sizes; meanwhile, in the soil, a uniform size is observed. The fact that the soil does not have a variety of sizes leads to an increase in the percentage of voids during its compaction, reducing its volumetric stability.

**Figure 2:**
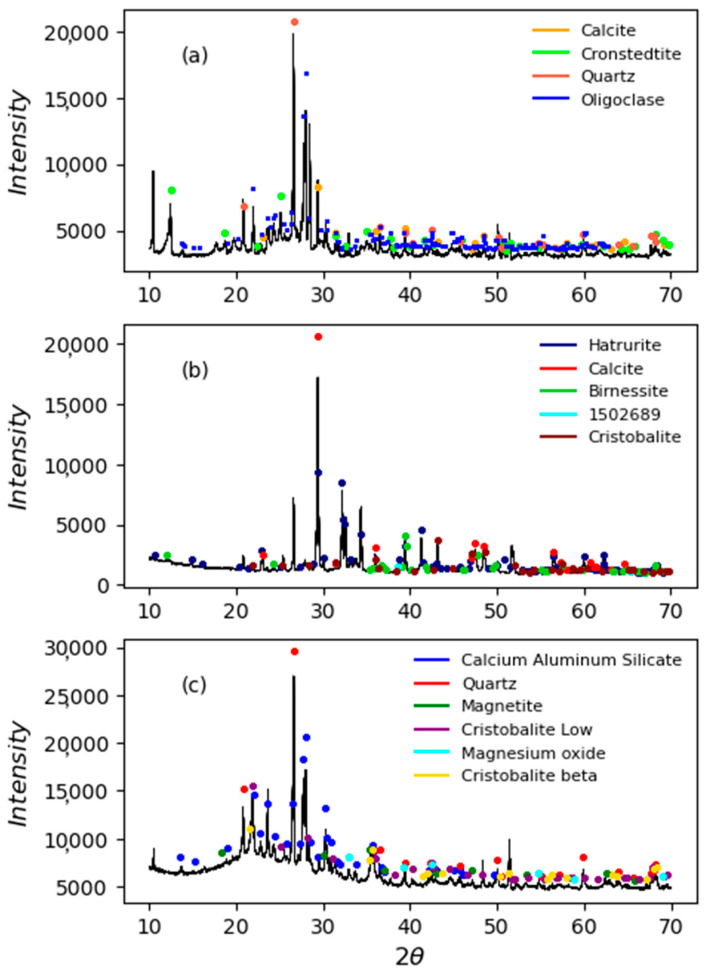
Diffractograms of the materials under study. (**a**) Soil, (**b**) ordinary Portland cement (OPC), and (**c**) sugarcane bagasse ash (SCBA).

Figure 2 shows the diffractograms of the soil, the OPC, and the SCBA obtained by X-ray diffraction (XRD) in a Panalytical Empyrean diffractometer. The main phases found in the soil were calcite (Ca_6_C_6_O_18_), cronstedtite (Fe_3.52_Si_1.48_O_9_H_4_), quartz (Si_3_O_6_), and oligoclase (Na_1.45_Ca_0.55_Al_2.55_Si_5.45_O_16_). In the case of OPC, the hatrurite (Ca_27_Si_9_O_45_), calcite (Ca_6_C_6_O_18_), birnessite (Mn_1_O_2_In), and cristobalite (Si_4_O_8_) phases were identified. In the SCBA, the phases identified were calcium aluminum silicate (Al_1.77_Ca_0.88_O_8_Si_2.23_), quartz (SiO_2_), magnetite (Fe_2.929_O_4_), low-cristobalite (SiO_2_), magnesium oxide (MgO), and beta-cristobalite (O_2_Si). In the case of the SCBA, amorphous material can be observed between angles 15 and 35 2θ, so a pozzolanic reaction can be expected.

**Figure 3:**
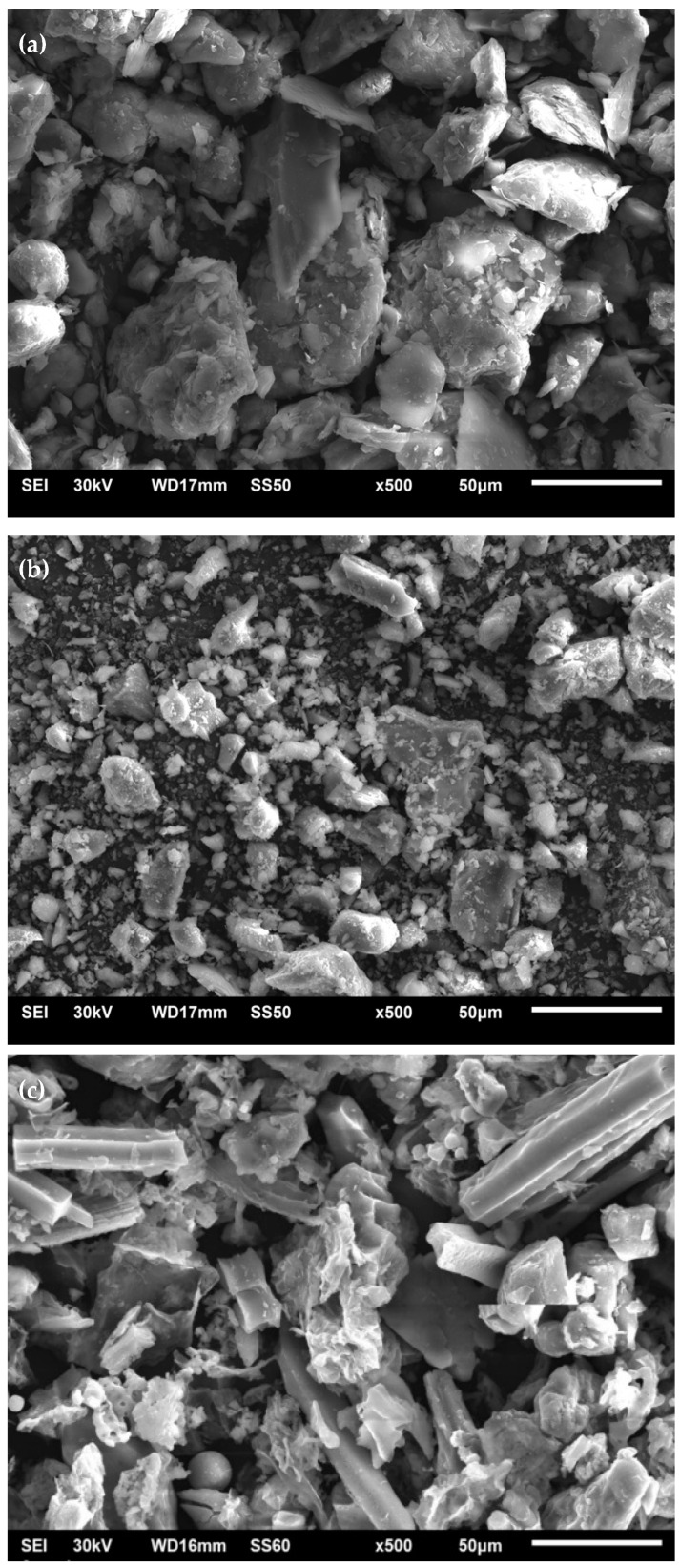
Micrographs at 500× of the materials under study: (**a**) Soil, (**b**) ordinary Portland cement (OPC), and (**c**) sugarcane bagasse ash (SCBA).

Figure 3 shows the morphology of the materials used obtained by JEOL 6510LV scanning electron microscopy (SEM) at a focus of 500× and a working distance of 16 mm with secondary electrons. A more irregular morphology can be observed in the SCBA. Prismatic particles with a defined structure are observed in the SCBA, generally rich in silica, spherical particles formed during combustion, and irregularly shaped particles. The soil shows a well-defined structure associated with its high silica content. In contrast, the morphology of the OPC exhibits variable shapes and a well-defined size distribution.

### 2.2. Sample Preparation

Table 3 shows the proportions used for the preparation of the soils with the different percentages of OPC replacement by SCBA and the combinations of these. Mixing was carried out for 1 min in the dry state, adding distilled water later until the optimum water content was reached per mixture. These were mixed for 3 min to hydrate the specimens and induce a reaction between the soil and the stabilizers. Subsequently, the mixtures were compacted to perform the compaction and CBR tests. Likewise, specimens were subjected to a curing process for compressive strength at a controlled temperature of 24 ± 1 °C for 28 days in the laboratory.

### 2.3. Test Methods

Particle size classification was used to characterize the natural subgrade soil through the standard test for granulometric analysis and the evaluation of the plasticity parameters using the procedures defined in the standard [33]. The Unified Soil Classification System (USCS) [38] was utilized to classify soils based on particle size, liquid limit, and plasticity index. Subsequently, the procedure established in the standard [34] was followed for the compaction test of the natural and stabilized soils, allowing a compaction curve to be obtained and determining the relationship between the optimum water content and the density of the stabilized and unstabilized soils. In compacted and saturated soils, the CBR test was performed following the procedures established in the standard [35]. Finally, the unconfined compressive strength was determined using the simple compression test according to the standard [31].

#### 2.3.1. USCS Classification

One of the fundamental aspects of the physical characterization of construction materials is their classification. For this reason, the size distribution of the natural subgrade soil particles was quantitatively determined using the standard test for granulometric analysis [32]. The separation of the particle sizes larger than 0.075 mm was carried out using the No. 200 sieve. Likewise, the percentage of fine soil with a particle size of less than 0.075 mm was quantified by taking the weight of the material particles on the scale that passed through the No. 200 sieve. For this last selection of fine material, its plasticity parameters were assessed using the procedures defined in the standard [33] to obtain the soil classification corresponding to the fine fraction.

For the classification of soils for engineering purposes, the USCS [38] has been taken as a reference, achieving soil classification by determining characteristics in the laboratory, such as particle size, liquid limit, and the plasticity index, with a precise scope of classification. The liquid limit and the plastic limit were determined, as these are required for the classification of fine soils according to the procedure [33], and for its execution, the fine fraction of the material was extracted using the No. 40 sieve.

#### 2.3.2. Compaction Test

The compaction of the natural and stabilized soils with the different proportions of OPC and SCBA shown in Table 3 was carried out according to the procedure defined in the standard [34]. The results obtained with the material evaluated at different moistures allowed plotting the compaction curve and obtaining a relationship between the optimal water content and the density of the stabilized and unstabilized soils. With the data obtained in this test method, the maximum dry unit weight and the optimal water content due to the preparation of the specimens with the different substitutions of SCBA and OPC were calculated.

#### 2.3.3. California Bearing Ratio (CBR) Test

The CBR parameters were measured on previously compacted and saturated soils using a load piston that penetrates the soil and is connected to a plotter where the load and the penetration depth are related. The initial graph is a curve with the initial section straight and the final section concave downwards. When the initial section is not straight, a correction is made. The load values supported by the soil when it sinks 2.5 mm and 5 mm are expressed as a percentage, and the highest percentage is used as the CBR index. The CBR value is not constant and depends on the density and water content of a given soil type. This procedure measures the load necessary to penetrate a compacted soil sample with a piston according to the procedures described in the standard [35], using the Humboldt brand multi-test machine, model HM-3000-3F (HUMBOLDT, 875 Tollgate Road Elgin, IL 60123, USA).

#### 2.3.4. Unconfined Compressive Strength

A simple compression test determined the resistance to uniaxial compression directly. This test method consisted of applying a load until the failure of the sample by using a press that exerts an axial force (F) at a load speed of 1 mm/min on a cylindrical specimen with a circular section and then calculating the resistance by utilizing the relationship between force over area. Sample preparation and the test procedure were carried out according to the standard [31]. The equipment used for the test was the Humboldt brand multi-test machine model HM-3000-3F, which has a maximum load capacity of 5 kN and a precision of 2.5 N. The equipment has sensors that record the data to be sent to the storage software. The samples were tested at a curing time of 28 days.

## 3. Results and Discussion

Table 4 summarizes the results obtained in the experiments carried out according to the experimental matrix of Table 3. The following sections describe the results of each test method in greater detail.

### 3.1. Effect on the Compaction of Stabilized Soil

**Figure 4:**
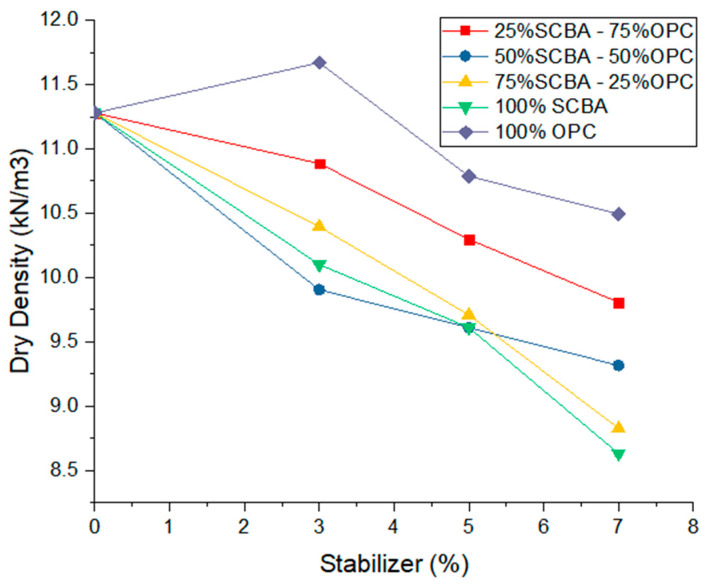
Maximum dry unit weight as a function of stabilizer percentage at different substitution percentages of ordinary Portland cement (OPC) by sugarcane bagasse ash (SCBA).

Figure 4 shows the effect experienced by the soil with the substitution of stabilizers on the maximum dry unit weight. In the first place, substituting 3% of cement without SCBA increases the maximum dry unit weight, while the percentage of optimal soil moisture decreases compared to the soil without a stabilizer. These variations are the product of the effect of the cement that hardens the soil particles with which it comes into contact, and as a consequence of this, the maximum dry unit weight of the stabilized soil increases. The increase in the unit weight with 3% cement as a stabilizer can be associated with three mechanisms: (1) There is greater compaction due to the good distribution of the particles [30]. (2) The tension due to the water suction in the pores produces an adhesion phenomenon between particles due to negative pressure or capillary forces, called apparent cohesion [31]. (3) The OPC hydration reaction happens early on due to the change in reaction kinetics [39,40,41].

**Figure 5:**
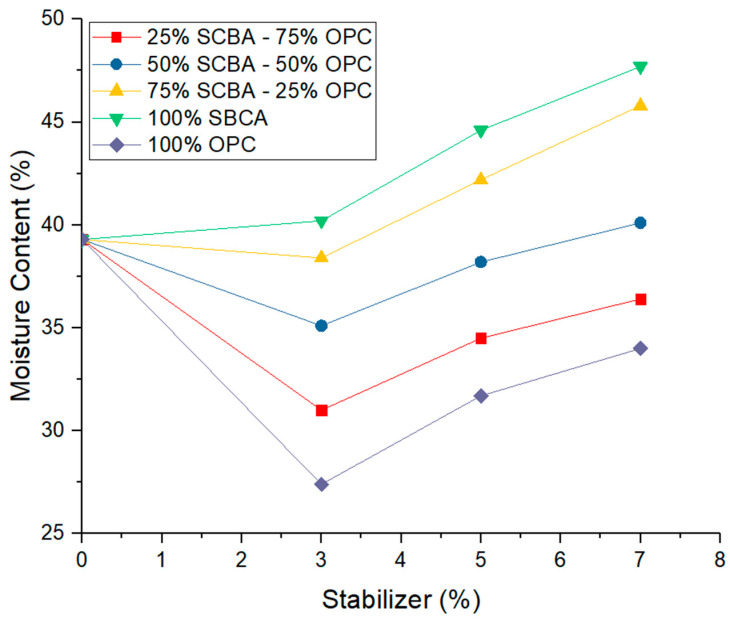
Optimum compaction moisture content as a function of stabilizer percentage at different percentages of replacement of ordinary Portland cement (OPC) by sugarcane bagasse ash (SCBA).

On the other hand, with the substitutions of 5% and 7% cement without SCBA, the maximum dry unit weight and the optimum compaction humidity decreased in relation to the soil parameters without a stabilizer, as can be observed in Figure 5. This shows that the chemical reaction of the minerals of the soil with the cement did not produce the optimal cementation, and for this reason, the voids inside the mixtures increased. In the same way, Figure 4 shows the effect of substituting the OPC and the SCBA in 3%, 5%, and 7% on the maximum dry unit weight of the soil, i.e., with the following dosages for each percentage of stabilizer: 75% OPC—25% SCBA and 50% OPC—50% SCBA. In these mixtures, the unit weight decreased slightly in relation to the soil stabilized with OPC. This occurs due to the physical characteristics of the SCBA, which has a low dry unit weight. Therefore, the percentage of OPC is replaced with a percentage of SCBA, decreasing the dry unit weight. The opposite situation was observed in the ascendent behavior of the required optimum humidity value, which increases proportionally to a factor of 1.1 with the replacement of OPC by SCBA compared to mixtures with 100% OPC. This tendency is caused by the need for hydration of the OPC and the absorption generated by the SCBA when incorporated into the mixture. These results coincide with what is indicated in the literature, where soils stabilized with OPC behaved similarly to those of the current study. Although there are changes in the percentages of optimum moisture and dry unit weight in the compaction tests, these variations are slight with respect to the soil without cement replacement. This is a characteristic required in soil stabilization, given that the effect of the cement will only be to improve the durability and resistance of the soil.

In the case of the soil with substitutions of 3%, 5%, and 7% stabilizer in the combinations of 25% OPC—75% SCBA and 100% SCBA, the highest decreasing changes in the dry unit weight values and increases in the optimum stabilized soil moisture percentage values were obtained. This is due to the higher percentage of SCBA that has a lower weight than the soil particles and the hydration it requires. Therefore, the light nature of the SCBA and higher porosity than the soil weight may be responsible for the decrease in the dry unit weight of the samples.

For the soils with a substitution combination of 50% OPC—50% SCBA with 3% of this arrangement, their unit weight showed a lower behavior but similar to the stabilization value with 100% OPC. This result can be associated with a suitable stabilizer percentage for this fine soil with high plasticity, which behaves adequately with the substitution of 50% of OPC. Likewise, with this dosage, the amount of water required for the absorption of the ash produces the disappearance of the forces that allow efficient compaction, decreasing the construction costs of the roads, as has been demonstrated in recent studies [42,43,44]. Soils with 100% SCBA also showed increases in the amount of water required and a significant decrease in their dry unit weight, with a greater range in the stabilizer percentages of 5% and 7%.

It is essential to highlight that the decrease in the percentage of optimal soil moisture with 3% stabilizer in the proportions of 50% OPC—50% SCBA can be generated by the water absorption capacity of the cement and verified with the substitution of stabilizer in 5% and 7% in the proportions described above. Although the increase is small, it remains below the value of the soil without a stabilizer [45]. Moreover, a decrease in the maximum dry unit weight of the stabilized soil is observed when substituting 3%, 5%, and 7% of the stabilizer in the proportions of 50% OPC—50% SCBA, which is because the SCBA occupied more space within the soil mixture, and this substitution generates a higher percentage of voids in the mixture given the porosity of the ash. Consequently, a decrease in the dry unit weight is registered.

### 3.2. Effect on the Unconfined Compressive Strength of the Stabilized Soil

**Figure 6:**
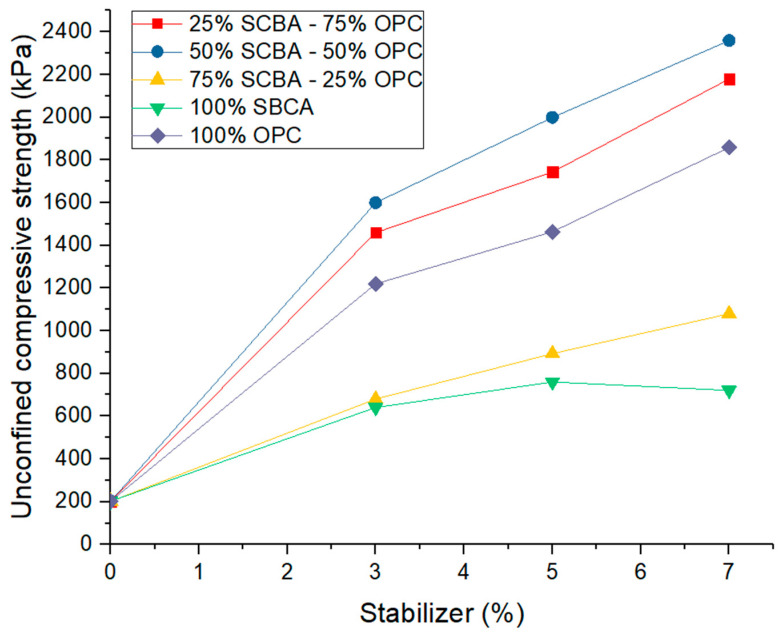
Unconfined compressive strength as a function of stabilizer percentage at different substitution percentages of ordinary Portland cement (OPC) by sugarcane bagasse ash (SCBA).

This analysis was carried out for the soil sample and the soil mixtures with the different stabilizer percentages during a curing period of 28 days. As expected, there is an improvement in the resistance to unconfined compression of the natural soil with the substitution of 3%, 5%, and 7% of OPC. Taking as a reference the value of 202 kPa obtained before the cement substitution, with 3%, 5%, and 7% OPC substitution, the results of the unconfined compression were 1219.7, 1463.8, and 1858.1 kPa, respectively, reaching more than 900% additional resistance. Figure 6 shows the resistance of the soil to simple compression with substitution of 3%, 5%, and 7% of the stabilizer comprised of different combinations of OPC and SCBA. Regarding the combination of 75% OPC—25% SCBA and 50% OPC—50% SCBA, a resistance similar to that registered with OPC substitutions without SCBA was observed. For the first combination, a 3% substitution showed an unconfined resistance of 1459.6 kPa; for a 5% substitution, the value was 1743.7 kPa; and for a 7% substitution, an unconfined resistance of 2179.4 kPa was registered. However, the result obtained in the second combination stands out, recording the best performance of the mixtures. An unconfined resistance of 1599.6 kPa was found for a 3% substitution, a value of 1999.5 was found for a 5% substitution, and an unconfined resistance of 2359.4 kPa was registered for a 7% substitution.

Correspondingly, results showing improved resistance of the soil with the combination of 25% OPC—75% SCBA and 0% OPC—100% SCBA were also obtained. In the first case, the resistance with a 3% stabilizer was 679.9 kPa; with a 5% stabilizer, the value exhibited was 892.9%; and for a 7% stabilizer, the value recorded was 1079.8 kPa. Lower values were obtained with the substitution of 100% SCBA. For this case, with the substitution of 3% SCBA, a resistance of 639.8 kPa was obtained; with the substitution of 5% SCBA, a resistance of 759.8 kPa was recorded, and for a 7% substitution of SCBA, a resistance of 719.8 kPa was observed. These results verify the stabilization that occurs in the soil and the 300% increase in the resistance to unconfined compression. However, when the SCBA content increases without some amount of cement, the compressive strength is the lowest because SCBA by itself does not show a cementitious value early on. On the other hand, according to the resistance results obtained with the combination of 50% OPC—50% SCBA, cement consumption can be reduced by up to 50%, obtaining similar and adequate resistance for a structural layer, optimizing the compaction process as discussed in Section 3.1, in addition to taking advantage of an agro-industrial residue whose storage generates a significant contamination problem. The improvement in the compressive strength in the combination of 75% OPC—25% SCBA and 50% OPC—50% SCBA is ascribed to avoid reduction as a consequence of the increase of size distribution promoted by the SCBA, leading to the increase in the surface area and contact points in the soil-cement matrix. During the curing period (28 days), the contact points develop a series of hydration products that are mechanically stable. Nevertheless, with higher SCBA content and lower OPC, the contact points are not enough to improve the stability of soil.

### 3.3. Effect on the California Bearing Ratio (CBR) of the Stabilized Soil

**Figure 7:**
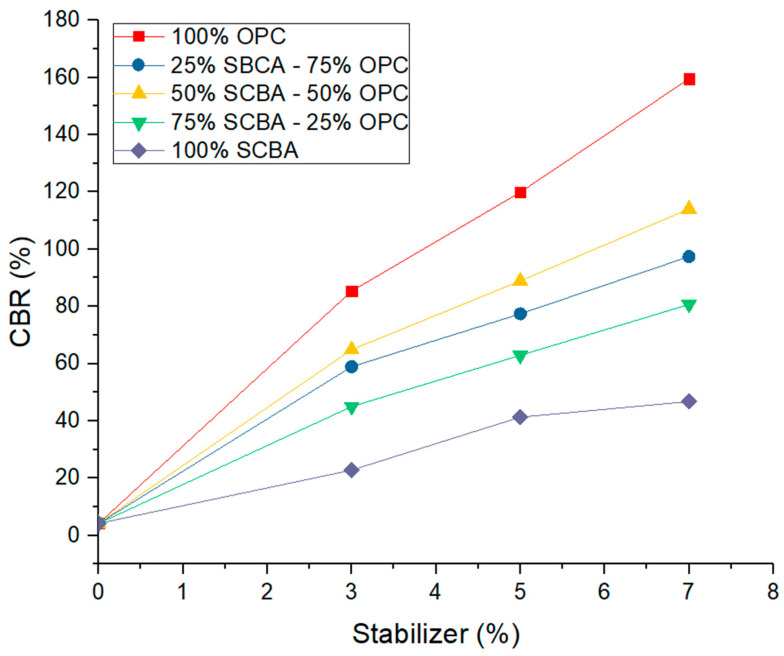
California bearing ratio (CBR) as a function of stabilizer percentage at different substitution percentages of ordinary Portland cement (OPC) by sugarcane bagasse ash (SCBA).

Figure 7 shows the behavior of the CBR in the different stabilized soil mixtures tested. In this figure, the different mixtures perform similarly to that presented in the simple compression resistance test. Likewise, the soil with substitution of 100% OPC presented significant increases in the CBR values compared to the soil without substitution, which has a CBR value of 4.20% and is taken as the reference. The soil with 3% OPC substitution showed a CBR of 85.40%; the soil with 5% OPC substitution reported a CBR of 119.9%, and when substituting 7% OPC, the soil reached a CBR of 159.6%.

Drawing parallels from previous research, [46] reported CBR increase values in a soil or sub-base type material similar to those obtained in the present investigation, with the soil including 3%, 5%, and 7% CP performing best, followed by the sub-base with the inclusion of a combination of 25% CBCA + 75% CP, with values higher than the soil without any addition.

On the other hand, the soils with substitutions of 3%, 5%, and 7% of the combination of 75% OPC—25% SCBA show good performance in the CBR test. The soil with 3% of this combination registered a CBR of 58.9%; the soil with substitution of 5% showed a CBR of 77.4%; and the soil with 7% substitution obtained a CBR value of 97.4%, the latter remaining below the soil with an OPC at 62.2% but with superior performance to the soil without substitutions in 93.2%.

In a related study, [47] assessed the impact of CBCA on the mechanical properties of a hydraulic base material for road construction and obtained notable increases in the CBR. The study soil with the highest increase compared to the behavior of the hydraulic base without the inclusion of Portland cement or CBCA was the soil with 3%, 5%, and 7% Portland cement, with an increase in its CBR by 33%, 44%, and 55% respectively. However, the hydraulic base with the inclusion of the combination of 75% CP + 25% CBCA, as well as the one with the inclusion of 50% CP + 50% CBCA, in the three study percentages of 3%, 5%, and 7%, increased their CBRs by 20%, 30%, and 40% compared to the soil without addition, proving that CBCA in combination with CP improves the mechanical properties of a hydraulic base type material.

For the stabilized soil sample with a combination of 50% OPC—50% SCBA, a 3% substitution registered a CBR of 64.9% associated with its excellent performance primarily due to the water-cement hydration reaction and to a lesser extent to the pozzolanic reaction and the contribution of SCBA to fill the pores of the mixture, increasing the contact area between particles and forming a rigid network. It should be noted that its performance is better than the dosage of the previous mixture. Thus, the combination of 50% OPC achieves the best behavior. The performance of the stabilized soils with a combination of 50% OPC—50% SCBA with a 5% substitution of stabilizer obtained a CBR of 88.9%, and the soil with 7% substitution showed a value of 114.1%, which is associated with the discussion carried out in Section 3.1 regarding the compaction process that occurs in the mixture mentioned above.

Further corroborating these findings, [48], when evaluating CBCA as an addition in igneous origin hydraulic base material, reported the same behavior as indicated by Landa (2017) but with increases of up to more than 100% with the addition of the combinations of 25% CP + 75% CBCA as well as with the combination of 50% CP + 50% CBCA, which was only evaluated with additions of 5% and 7%. This suggests that its implementation in road construction would impact the reduction of thicknesses of the hydraulic base layer, in addition to the use of the agro-industrial waste contributing to the environment and a sustainable development of our society.

All the CBR values of the stabilized soils in the different dosages of OPC and OPC-SCBA significantly improve the mechanical characteristics of the subgrade material with conditions that will contribute in the design to the reduction of the thicknesses of the subbase and base layers for the pavement structure, which translates into a considerable reduction in the use of materials with a sustainable approach and a contribution to reducing the environmental impact of the construction and reconstruction of roads [49].

Concerning the soil with substitutions of 3%, 5%, and 7% at a combination of 25% OPC—75% SCBA, these showed better performance on the CBR test compared to the unstabilized soil. Specifically for the soil with 3% of this combination, the CBR result was 45.0%; the soil with the substitution of 5% registered a value of 62.9%; and the soil with 7% of this combination obtained a CBR value of 80.7%, with the latter remaining below the one of the soil with an OPC at 78.9% but with a performance above the one of the soil without substitutions at 76.5%. These results are associated with the water-cement hydration process, plus the reaction kinetics between Ca(OH)_2_ and the amorphous silica present in the SCBA observed in the XRD (Figure 2), which adopts changes from the first hours of mixing, as has been reported in previous investigations [50,51], in addition to a filling effect of the SCBA particles given its good granulometric distribution observed in Figure 2. These results are comparable with the results of various studies that evaluated the stabilization of residual soil with OPC and rice husk ash (RHA), finding that the maximum increase in CBR was achieved by substituting 4% OPC and 5% RHA. Further, the authors demonstrated, with the use of DRX and SEM, a change in the soil structure due to the pozzolanic reaction [52]. In another study, the stabilization of a CH-type soil (high plasticity clay) with substitution of OPC and RHA was evaluated, where the authors demonstrated that the optimal substitution of RHA was 10% with 6% of OPC to obtain the highest increases in resistance to simple compression and CBR [49].

Finally, with the CBR results of the current study, in the substitution percentages of 3%, 5%, and 7% of SCBA without cement substitution, with values of 22.8%, 41.3%, and 46.8%, respectively, significant increases in CBR with respect to soil without stabilizer were registered. These results are associated with the contribution of the SCBA as a filler to the soil and the compaction energy given to it. We show with these results that in confined conditions, the SCBA is more effective than in unconfined conditions like compressive strength. Better stability is reached during compaction, and the performance of the soil with regard to penetration load is improved, unlike a natural soil.

The CBR data show that using OPC as a stabilizer without SCBA substitution produces higher CBR values than the mixture of OPC and SCBA at all substitution percentages. This suggests that pure OPC is the most effective option to improve the bearing capacity of the soil.

However, it is essential to consider that applying the mixture of OPC and SCBA significantly improves soil resistance, obtaining CBR values with results higher than the limits established by most of the current international regulations. This demonstrates that the mixture of OPC and SCBA can represent a viable alternative in soil stabilization under a sustainable approach due to the availability of resources and the decrease in project investment costs of construction, complying with the mechanic requirements.

The incorporation of SCBA in the mixture improves the CBR values obtained, highlighting that despite not generating a cementitious process, the increase in CBR is due to the granulometric adjustment and consequently a reduction of voids in the matrix, offering additional advantages, such as the reduction in OPC consumption and the reuse of an industrial by-product, which favors construction and reduces environmental impact. In addition, using the OPC and SCBA mix could generate savings in construction costs due to the lower demand for OPC, which is generally a more expensive material.

## 4. Conclusions

The mechanical properties of a soil stabilized with ordinary Portland cement with significant substitution of sugarcane bagasse ash under a sustainable approach were analyzed. Three percentages of OPC as a stabilizing agent were considered, and the influence of three percentages of substitution of OPC by SCBA on the mechanical properties was analyzed. Based on the results of the formulations under study, the following conclusions were obtained:

The soils with partial replacement of OPC by SCBA in the percentages of 25%, 50%, and 75% in the three percentage of OPC reduced their maximum dry density, their unconfined compressive strength, and their California bearing ratio, with an increase in the amount of water needed to reach their maximum dry density.

The soils with partial replacement of OPC by SCBA in the percentages of 25%, 50%, and 75%, compared with the soil without substitutions, decreased their maximum dry density. However, their compressive strength and their California bearing ratio improved significantly.

The observed differences in the mechanical behavior of the test mixtures, according to the unconfined compressive strength response and the California bearing ratio, are attributed to differences in confinement conditions. While there is no geostatic pressure condition in the compressive strength, the California bearing ratio value allows the filler effect promoted by the SCBA to be more efficient through confinement. Therefore, partial substitutions of 25%, 50%, and 100% of OPC by SCBA increase the support value of the soil for subgrade.

The 25% partial replacement of OPC by SCBA shows the best performance in its maximum dry density, reaching a compressive strength and California bearing ratio significantly close to the soil values with 100% OPC; this could imply, in practical terms, a reduction in cement consumption.

There is a viable solution for SCBA waste management with an end use in soil-stabilizing materials that meets the mechanical requirements for use in subgrade layers in pavements.

## Figures and Tables

**Table 1:** Geotechnical properties of the soil.

Property	Standard Test Method	Value
Soil classification (USCS) [38]	ASTM D 422, 2007	MH
Liquid limit (%)	ASTM D 4318, 2010	83.21
Plastic limit (%)	ASTM D 4318, 2010	51.11
Plasticity index (%)	ASTM D 4318, 2010	32.10
Dry unit weight (kN/m^3^)	ASTM D 1557, 2009	11.28
Optimum moisture content (%)	ASTM D 1557, 2009	39.3
California bearing ratio (%)	ASTM D 1883, 2007	4.2

MH: elastic silt.

**Table 2:** Chemical composition of the soil, the ordinary Portland cement (OPC), and the sugarcane bagasse ash (SCBA) obtained by X-ray fluorescence (XRF).

Compound	% of the Total Weight		
	Soil	OPC	SCBA
SiO_2_	52.11	17.99	75.74
Al_2_O_3_	19.85	3.88	4.09
Fe_2_O_3_	10.73	4.76	8.55
CaO	7.21	62.28	2.88
MgO	4.57	1.71	0.50
P_2_O_5_	-	--	0.63
Na_2_O	0.76	0.23	1.22
SO_3_	-	4.03	0.41
K_2_O	2.44	--	3.88
TiO_2_	1.45	--	0.95
MnO	-	--	0.38
Density (g/cm^3^)	1.15	3.15	2.2
Specific Surface área (cm^2^/g)		4390	850

The values obtained are very low, or the presence of these compounds was not detected (-).

**Table 3:** Experimental matrix.

Mixture No	Soil	OPC	SCBA	% SCBA
Mixture 1	100	0	0	0
Mixture 2	97	3	0	
Mixture 3	95	5	0	
Mixture 4	93	7	0	
Mixture 5	97	2.25	0.75	25
Mixture 6	95	3.75	1.25	
Mixture 7	93	5.25	1.75	
Mixture 8	97	1.5	1.5	50
Mixture 9	95	2.5	2.5	
Mixture 10	93	3.5	3.5	
Mixture 11	97	0.75	2.25	75
Mixture 12	95	1.25	3.75	
Mixture 13	93	1.75	5.25	

OPC: ordinary Portland cement, SCBA: sugarcane bagasse ash.

**Table 4:** Summary of the results obtained in the soil stabilized with mixtures of ordinary Portland cement (OPC) and sugarcane bagasse ash (SCBA).

Mixture		Dry Unit Weight (kN/m^3^)	Moisture (%)	CBR (%)	Unconfined Compressive Strength (kPa)
OPC%	SCBA%				
0.00	0.00	11.28	39.3	4.20	202.0
3.00	0.00	11.55	27.4	85.4	1219.7
5.00	0.00	10.90	31.7	119.9	1463.8
7.00	0.00	10.75	34.0	159.6	1858.1
2.25	0.75	10.90	31.0	58.9	1459.6
3.75	1.25	10.51	34.5	77.4	1743.7
5.25	1.75	10.32	36.4	97.4	2179.4
1.50	1.50	9.89	35.1	64.9	1599.6
2.50	2.50	9.30	38.2	88.9	1999.5
3.50	3.50	8.81	40.1	114.1	2359.4
0.75	2.25	10.40	38.4	45.0	679.9
1.25	3.75	9.71	42.2	62.9	892.9
1.75	5.25	9.50	45.8	80.7	1079.8
0.00	3.00	10.19	40.2	22.8	639.9
0.00	5.00	9.61	44.6	41.3	759.8
0.00	7.00	9.41	47.7	46.8	719.8

CBR: California bearing ratio.

## Data Availability

The data are available from the leading author Pedro Julian Gallego Quintana upon request.
